# Polyunsaturated fatty acid relatively decreases cholesterol content in THP-1 macrophage-derived foam cell: partly correlates with expression profile of CIDE and PAT members

**DOI:** 10.1186/1476-511X-12-111

**Published:** 2013-07-23

**Authors:** Yue Song, Li-Jun Zhang, Hang Li, Yu Gu, Fan-Fan Li, Li-Na Jiang, Fang Liu, Jing Ye, Qing Li

**Affiliations:** 1 State Key Laboratory of Cancer Biology and Department of Pathology, Xijing Hospital, Fourth Military Medical University, Xi’an, China; 2 Department of Clinical Laboratory, Tangdu Hospital, Fourth Military Medical University, Xi’an, China; 3 Orthopedics Oncology Institute of Chinese People’s Liberation Army and Department of Orthopaedics, Tangdu Hospital, Fourth Military Medical University, Xi’an, China; 4 Hyperbaric Oxygen Center of Chinese People’s Liberation Army, Navy General Hospital, Beijing, China

**Keywords:** Polyunsaturated fatty acid, CIDE, PAT, Lipid metabolism, Atherosclerosis

## Abstract

**Background:**

Polyunsaturated fatty acids (PUFAs) have positive effect on the regulation of plasma lipids. But the mechanism for them to modulate lipid homeostasis in macrophage is still unclear. In this study, we employed PUFA to pretreat macrophages and evaluated the variations of lipid droplet (LD) content, lipid composition, and expressions of LD-associated genes in macrophage-derived foam cells.

**Method:**

THP-1-derived macrophages or human peripheral blood monocyte-derived macrophages were pre-treated with four non-esterified fatty acids (NEFAs) separately: saturated fatty acid (SFA)-palmitic acid (PA), monounsaturated fatty acids (MUFAs)-oleic acid (OA), PUFAs-linoleic acid (LA) and eicosapentaenoic acid (EPA). Intracellular lipid content and cholesterol efflux were analyzed in THP-1 macrophage-derived foam cells. Related gene expressions were detected by quantitative real-time PCR.

**Results:**

PUFA pre-treatment reduced cholesterol content in foam cells and increased cholesterol efflux to lipid-free apoAI in conditioned medium compared with PA or OA group. Cell death-inducing DFF45 like effector (CIDE) and Perilipin-Adipophilin-TIP47 (PAT) family members, as LD-associated proteins, showed specific gene expression profiles after PUFA pre-treatment. These results may help to explain the process of lipid metabolism within foam cells.

**Conclusion:**

PUFA (LA or EPA) had a potential protective effect against cholesterol accumulation. The specific expressions of CIDE and PAT genes may provide clues to explore the protective mechanism of PUFA in foam cells.

## Background

PUFAs are essential components in lipid metabolism. It has been reported that PUFAs can affect lipid storage and reduce the risk factors of cardiovascular diseases [1]. However, the potential role of PUFA in the formation of fatty streak and atherosclerotic plaque is not yet clear. The molecular mechanism for PUFA-induced lipid influx and efflux in foam cells is inconclusive.

Abnormal lipid deposition in the intima of artery wall leads to the formation of fatty streak. Macrophages take-up the oxidized LDL (ox-LDL), stores a large portion of lipids in their cytoplasm, and turn into foam cells [2,3]. The formation, morphology and lipolysis of intracellular LDs are regulated by a number of LD-associated proteins [4]. These proteins are located on the surface of LDs and play active roles in the regulation of intracellular lipid storage, nascent LD biogenesis and transportation [5-8]. CIDE and PAT proteins are by far the most specific LD-associated proteins being found. Proteomic analyses also confirmed that the most abundant proteins in LDs were the CIDE and PAT family members [9]. The peroxisome proliferator-activated receptor γ (PPARγ) is considered as an important transcription factor involved in the regulation of gene expression of most of LD-associated proteins [10-12]. Microarray analysis in our previous study [8] showed that CIDE and PAT members, together with PPARγ regulated proteins, had meaningful expression changes in the process of THP-1 macrophage-derived foam cells formation. These data suggested that CIDE or PAT proteins were closely related to intracellular LD formation. But different NEFA-induced protein expressions are still not clear.

In this study, THP-1-derived macrophages and peripheral blood monocyte-derived macrophages were pre-treated separately with PA, OA, LA and EPA (difference in saturation). We intend to simulate a microenvironment of monocyte-derived macrophages in artery wall. The lipid composition of intracellular LDs was identified by quantitative analysis in two cell groups separately. By analyzing cholesterol efflux and mRNA expression profiles of CIDE, PAT and PPARγ transcriptional regulatory proteins in THP-1 macrophage-derived foam cells, we try to present a potential relationship among PUFA pre-treatment, lipid loading and LD-associated gene expression.

## Results

### Identification of the optimum incubation condition

**Figure 1:**
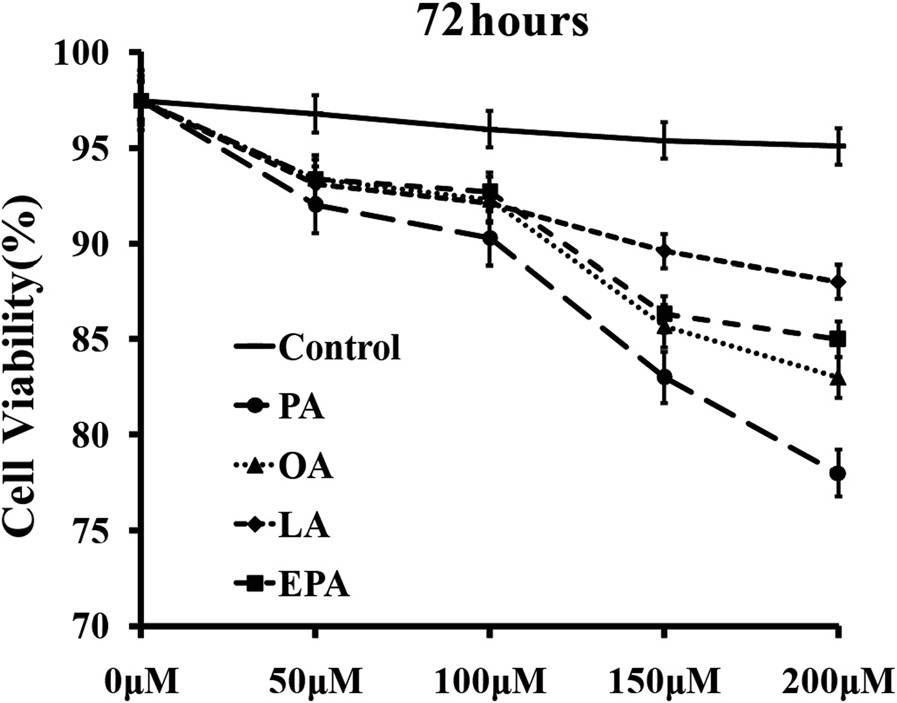
**Cell viability of THP-1 macrophage after pre-treatment with different NEFAs at different concentrations for 72 hours.** The THP-1-derived macrophages were pretreated by PA, OA, LA and EPA separately with time and dose gradients. The cell viability was determined by MTT colorimetric assay. Data represent mean ± SEM (n = 3).

Before the experiment, we examined the cytotoxicity of employed fatty acids on THP-1 macrophages with MTT assay. NEFA pre-treatment increased cell death rate in a time- and dose-dependent manner (Figure 1). Compared with the control group, 48 hours’ incubation by each NEFA had no effect on macrophage viability. While after 72 hours’ incubation, there were at least 75% cells survived in each group, which is sufficient for the following experiments. Concentration of NEFAs can also affect macrophage viability. Cell survival rate decreased slightly after 72 hours pre-treatment with 100 μM NEFAs. However, when the concentration was elevated to 200 μM, all the NEFAs markedly decreased cell viability (*P* < 0.05 separately). Therefore, we chose 100 μM and 72 hours as the optimum concentration and time condition for macrophages incubation in the following study.

### PUFA induced relatively lower lipid content in foam cells

**Figure 2:**
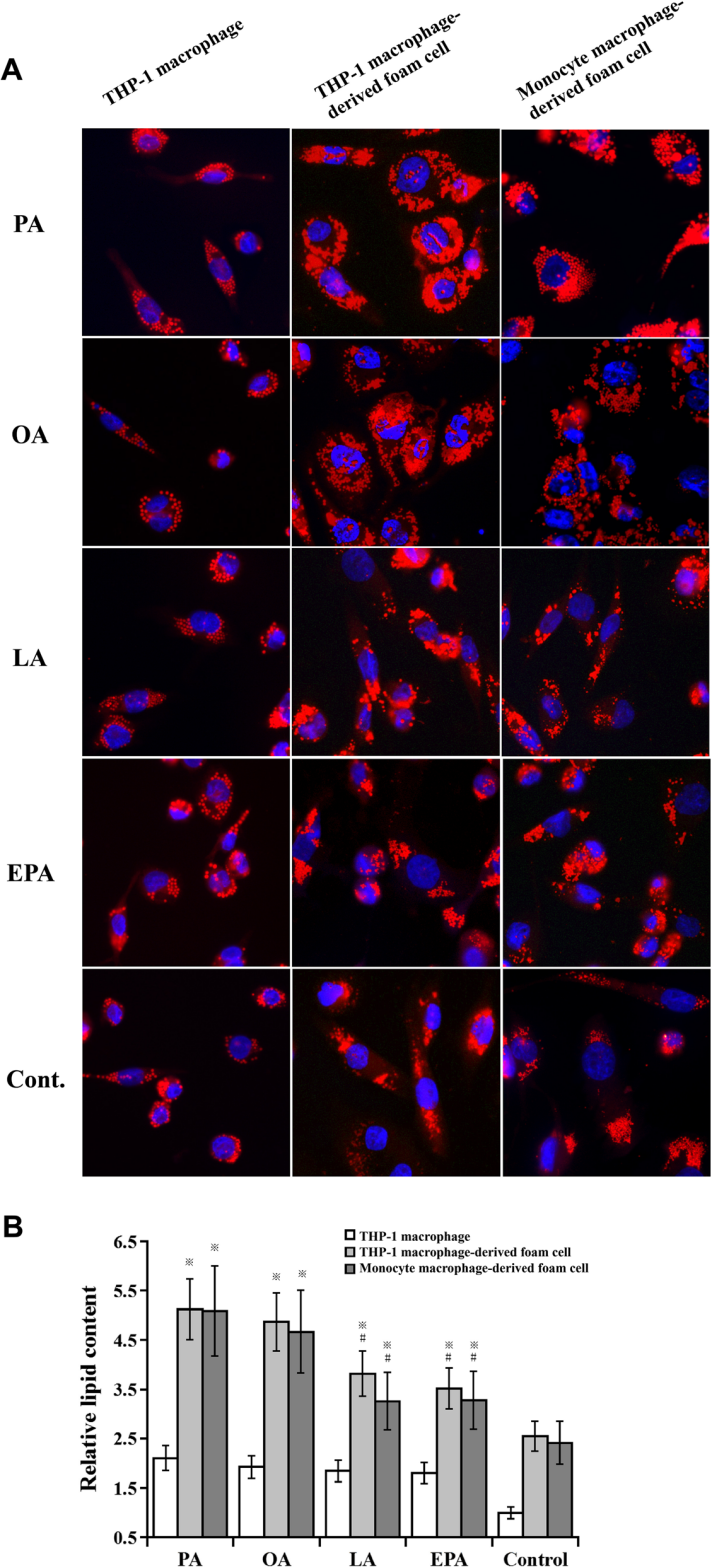
**Effects of PUFAs on intracellular lipid accumulation.** Both THP-1 macrophages and monocyte macrophage were pre-treated with different NEFA at the concentration of 100 μM for 72 hours. Then ox-LDL was added to facilitate foam cell formation. The intracellular lipid droplets were stained with Nile Red **(A)**. The average positive area of stained LDs per cell was used to quantify the intracellular lipid content **(B)**. Data represent mean ± SEM (n = 3). ^※^*P* < 0.05 vs. control; ^#^*P* < 0.05 vs. PA/OA group.

THP-1-derived macrophages were separately exposed to different NEFAs in advance for 72 hours. Then ox-LDL was added to facilitate the formation of foam cells. Intracellular LDs were stained with Nile Red (Figure 2A). And the LD content was quantified by measuring the positive area in macrophages and foam cells (Figure 2B). Due to the intake of ox-LDL, foam cells in the control group contained more LDs compared with non-ox-LDL treated THP-1 macrophages. All the four NEFAs could efficiently increase the total area of LDs in foam cells compared with control (*P* < 0.05). Lipid content in PUFA group was lower than that in PA and OA group (*P* < 0.05). To verify the effect of pre-treatment of PUFA, we then incubated human peripheral blood monocyte-derived macrophages with different NEFAs separately. The quantitative analysis of lipid content in foam cells was consistent with that in THP-1 macrophage-derived foam cells (Figure 2A-B). These findings illustrated that, compared with PA and OA, PUFAs (LA and EPA) could cause relatively less lipid content during foam cells formation.

### PUFA relatively inhibited cholesterol accumulation in foam cells

**Figure 3:**
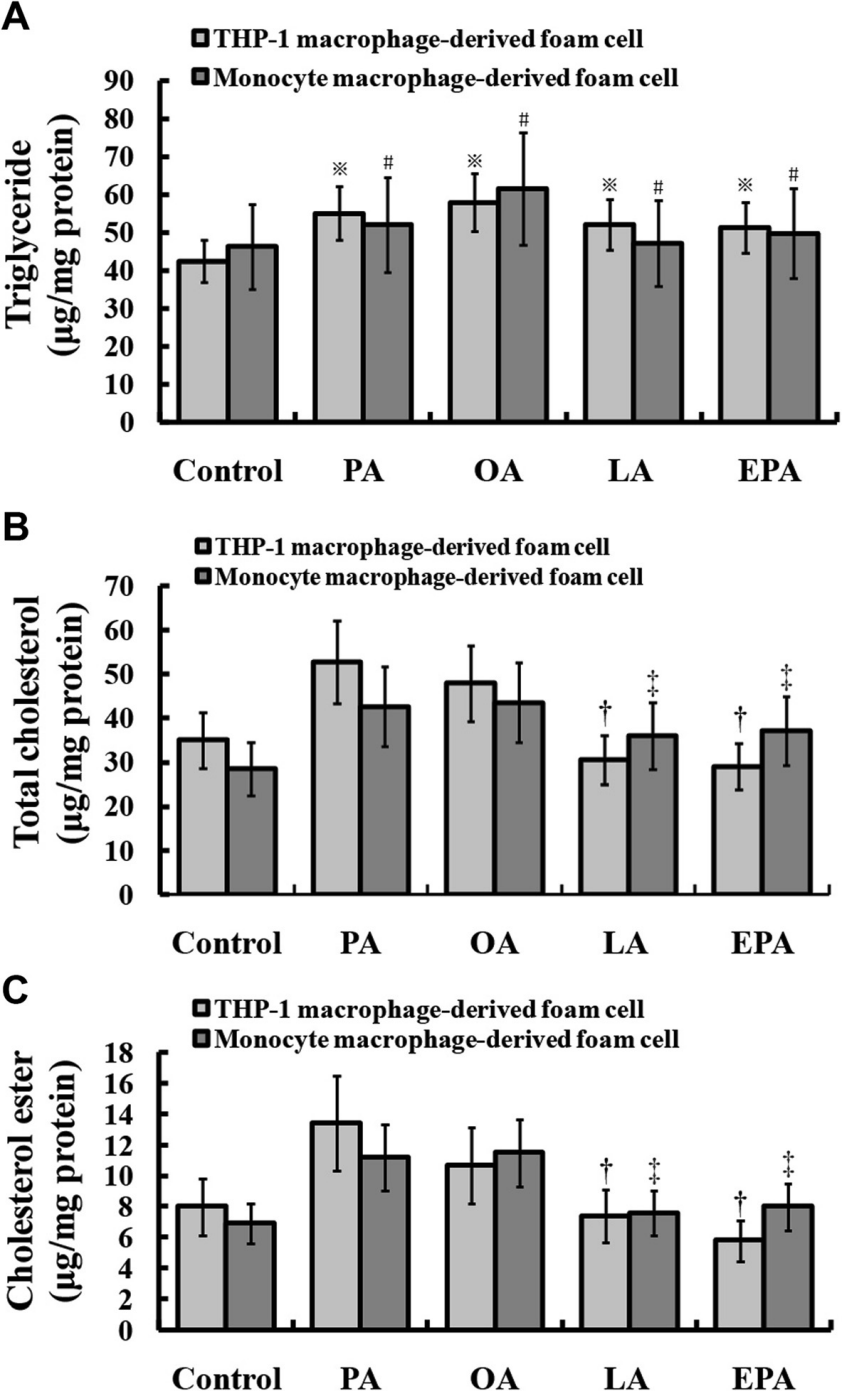
**Quantitative determination of TG, TC and CE in foam cells.** The THP-1 macrophages or monocyte macrophages were pre-treated with different NEFA in advance, and then ox-LDL was added to facilitate foam cell formation. Intracellular TG, TC and free cholesterol were determined by enzymatic colorimetric assays **(A**-**B)**. The concentration of CE was determined by subtracting free cholesterol from TC **(C)**. Data represent mean ± SEM (n = 3).^※/#^*P* < 0.05 vs. control **(A)**; ^†/‡^*P* < 0.05 vs. PA/OA group **(B**-**C)**.

We measured intracellular TG, TC and CE levels in both THP-1 macrophage- and monocyte macrophage-derived foam cells. Each NEFA could elevate the level of TG compared with respective control (*P* < 0.05, Figure 3A). PUFA induced less accumulation of TC and CE than PA or OA did during the process of foam cell formation (*P* < 0.05 respectively, Figure 3B-C). These results indicated that the lower lipid content in PUFA pre-treated group was partly due to less cholesterol accumulation.

### PUFA increased cholesterol efflux to lipid-free apoAI in conditioned medium

**Figure 4:**
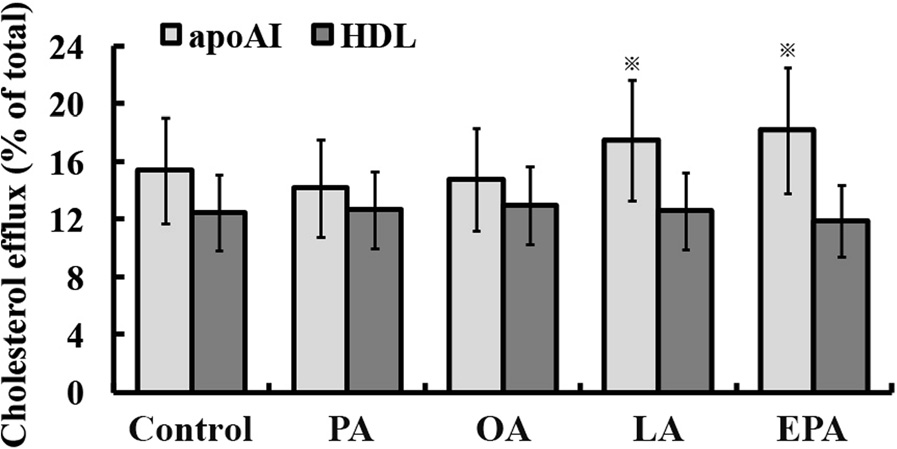
**Effects of PUFAs on cholesterol efflux to lipid-free apoAI or to HDL.** THP-1 macrophages or monocyte-macrophages were pre-treated with different NEFA, and then incubated with ox-LDL containing 1 μCi/ml [3H] cholesterol and 2 mg/ml BSA. The radioactivity was measured by scintillation counting. Cholesterol efflux to apoAI from foam cells was increased in LA and EPA groups. PUFAs had no effect on cholesterol efflux to HDL from foam cells. Data represent mean ± SEM (n = 3). ^※^*P* < 0.05 vs. control.

To test whether the lower intracellular cholesterol accumulation was caused, at least partly, by increased cholesterol efflux, we performed cholesterol efflux assays. We detected the amount of cholesterol efflux from THP-1 macrophage-derived foam cells to either lipid-free apoAI or to HDL in medium. There was no statistical significance in cholesterol efflux to HDL among NEFA groups. But cholesterol efflux to lipid-free apoAI was markedly increased in PUFAs (LA and EPA) groups compared with that in PA or OA group (Figure 4).

### The mRNA levels of PPARγ transcriptional regulated proteins were closely correlated with intracellular lipid content

**Figure 5:**
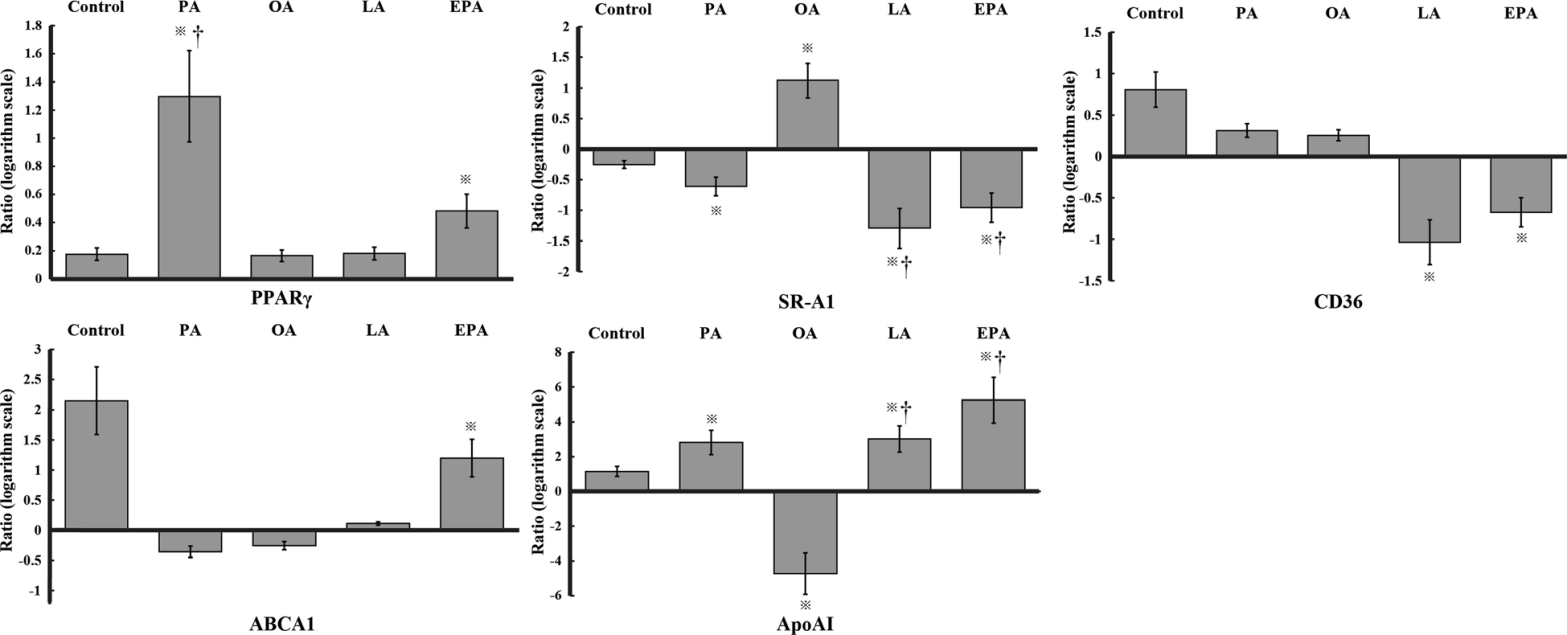
**Effects of PUFAs on the mRNA expressions of PPARγ transcriptional regulated proteins during THP-1 macrophage-derived foam cell formation.** The THP-1 macrophages were pre-treated with different NEFA in advance, and then ox-LDL was added to facilitate foam cell formation. Total RNAs were extracted from both macrophages and foam cells and reverse-transcribed into cDNA. PPARγ, SR-A1, CD36, ABCA1 and apoAI were measured by real-time PCR. Data represent mean ± SEM (n = 3). ^※^*P* < 0.05 vs. the value in macrophages; ^†^*P* < 0.05 vs. the un-treated control.

PPARγ is a critical nuclear receptor regulating the expression of LD-associated proteins [6], part of which have been found having specific expression profiles during foam cell formation [8,12]. In our previous microarray analysis, we found that the expression of SR-A1, CD36, ABCA1 and apoAI showed interesting changes. In this study, we tried to clarify potential roles of these genes in modulating cellular lipid loading after PUFA pre-treatment. Data in Figure 5 and Table 1 directly showed fold-changes of related mRNAs in foam cells vs. in macrophages. Specific NEFA induced mRNA expression on macrophages and foam cells were shown in Additional file 1: Figure S1 and Additional file 2: Figure S2.

**Table 1:** Specific NEFA induced fold-changes of mRNAs

**Protein**	**Control**	**PA**	**OA**	**LA**	**EPA**
SR-A1	-0.25	-0.61*	1.12*	-1.30*^#^	-0.96*^#^
CD36	0.81	0.32	0.26	-1.03*	-0.67*
ABCA1	2.15	-0.36	-0.26	0.11	1.20*
ApoAI	1.15	2.82*	-4.73*	3.02*^#^	5.24*^#^
PPARγ	0.18	1.30*^#^	0.17	0.18	0.48*
Cidea	0.02	-1.54*	-3.87*	3.17*^#^	1.02^#^
Cideb	1.35	2.43*	1.67*	0.28^#^	0.17^#^
Cidec	0.65	-0.24	-0.27	0.84^#^	1.75*^#^
Perilipin	-0.15	-2.11*	-3.04*	2.42*	-0.24
ADRP	4.05	5.22*	4.36*	9.55*^#^	7.78*^#^
TIP47	0.99	1.39*	0.54*	-1.79*	2.04*^#^
S3-12	0.38	2.10*	-2.80*	6.30*^#^	3.21*^#^
LSDP5	1.03	1.60*	-2.02*	2.96*^#^	0.10

Values were presented as: log_2_ (mRNA level in foam cell/mRNA level in macrophage).

(*P < 0.05; ^#^*P* < 0.05 vs. respective control).

PPARγ can be activated by some long-chain saturated or unsaturated fatty acids. We found that PUFA separately decreased the expression of SR-A1 and CD36, while increased the expression of apoAI in foam cells compared with PA or OA. PUFA also induced notable fold-increase of apoAI compared with the untreated foam cell control. ABCA1 was markedly up-regulated by EPA but not by LA. The expression profiles of PPARγ related proteins induced by PUFAs were, to a great extent, consistent with lower cholesterol accumulation and higher cholesterol efflux. SFA and MUFA neither showed protective effect against lipid loading nor induced similar expressions of related genes as PUFAs in foam cells.

### Expression profiles of CIDE and PAT members were correlated with PUFAs induced LD formation

Our previous study has shown that LD-associated proteins have specific expression profiles in the process of foam cell formation. In this study, we further investigate NEFA-induced alterations of CIDE and PAT members in foam cells.

**Figure 6:**
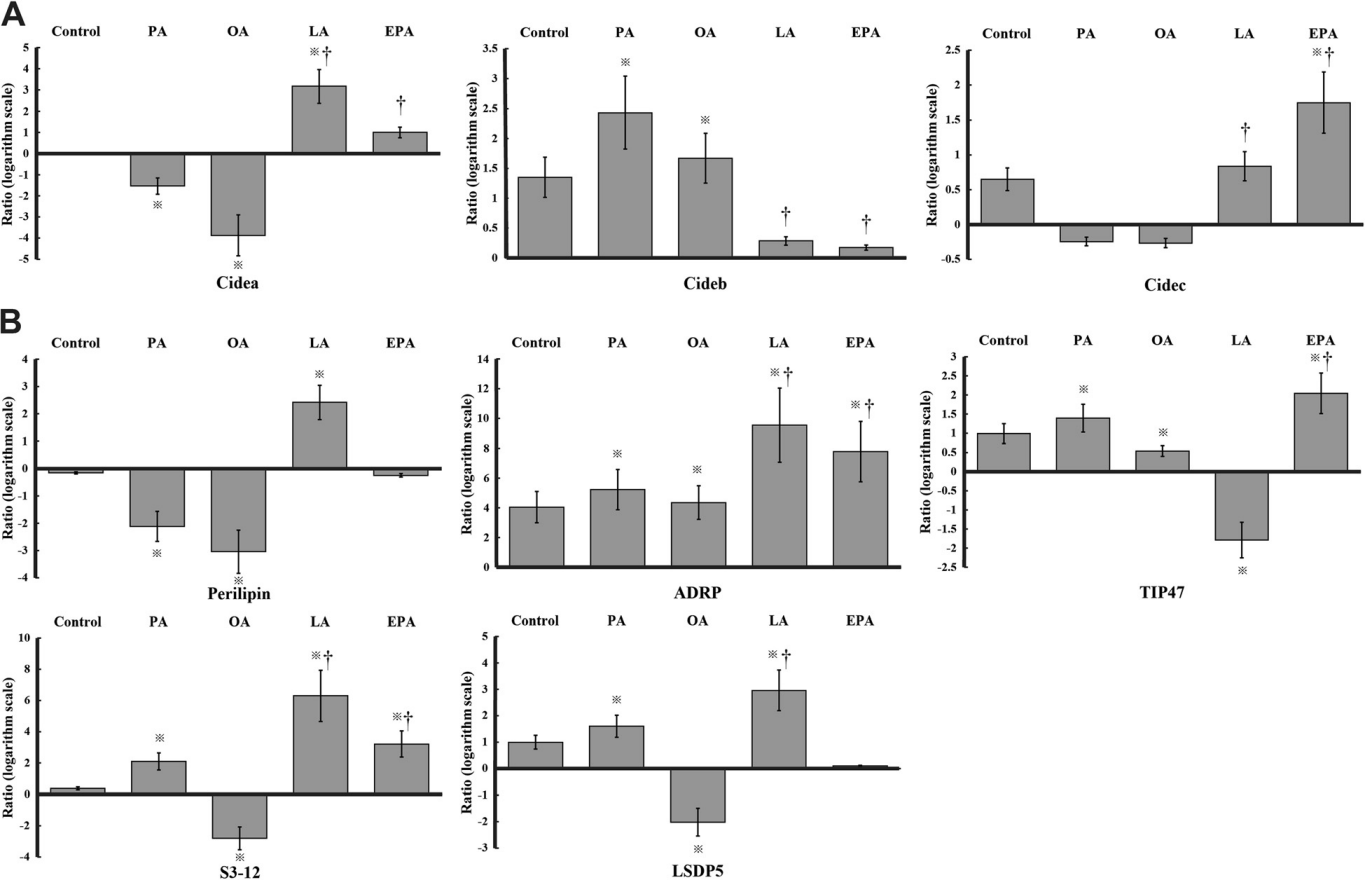
**Effects of PUFAs on the mRNA expressions of CIDE and PAT members during NEFA-induced LD formation.** The THP-1 macrophages were pre-treated with different NEFA in advance, and then ox-LDL was added to facilitate foam cells formation. Total RNAs were extracted from both macrophages and foam cells and reverse-transcribed into cDNA. CIDE **(A)** and PAT **(B)** family members were measured by real-time PCR. Data represent mean ± SEM (n = 3). ^※^*P* < 0.05 vs. the value in macrophages; ^†^*P* < 0.05 vs. the un-treated control.

As shown in Figure 6A and Table 1, PUFA significantly up-regulated the expression of Cidea and Cidec while reduced the expression of Cideb compared with the foam cell control. PAT family members have been proved to be the dominant LD-associated proteins. NEFA-induced fold-changes of PAT members in transcriptional level were showed in Figure 6B and Table 1. ADRP, a protein which has similar function with Perilipin, increased remarkably by each NEFA pre-treatment. ADRP showed greater fold-increase in PUFA groups compared with it in PA or OA group. Interestingly, of all employed NEFAs, LA induced the highest fold-increase of PAT members except for TIP47, and EPA similarly induced significantly fold-increase of PAT members except for Perilipin. These specific gene expression profiles, combined with their functions in the process of LD formation, provided a relevant explanation to the alterations of intracellular lipid content.

## Discussion

Numerous studies show that PUFAs play potential roles in maintaining serum lipid homeostasis. Population-based study found that high level of fish intake was good for cardiovascular health [13]. PUFA had been reported to be a protective agent against atherosclerosis (AS) [14,15]. In this study, we chose different NEFAs according to their importance in human diet. NEFAs are rich in meat (PA), plant fats (OA, LA), and fish oils (EPA *n*-3). PA (16:0), OA(18:1 *n*-9), and LA(18:2 *n*-6) are the most abundant fatty acids in fatty streaks of human [16]. Our study confirmed that compared with SFA and MUFA, PUFAs (LA and EPA) could relatively reduce intracellular TC and CE, thus lower the total lipid content in foam cells. However, NEFAs were stored as TGs in budding LDs and ox-LDL lipids were added to the pre-existing ones, which increased sizes of LDs finally. This helps to explain the theory that PUFA promotes the storage of TG.

NEFAs regulate gene expressions by acting directly or indirectly as ligands for nuclear acceptors [17]. PPARγ binds DNA in the form of heterodimer with retinoid X receptor (RXR) and maintains lipid homeostasis in macrophages by regulating the expression of scavenger receptors including SR-A1 and CD36 [5]. Meanwhile, PPARγ is the upstream regulator of ATP-binding cassette transporter ABCA1 and apolipoprotein AI (apoAI). The classic pathway of cholesterol efflux from macrophages is that CD36 or SR-A1 on the surface of macrophages recognizes and engulfs ox-LDL, and then ABCA1 exports cholesterol and phospholipids to lipid-free apoAI, forming small HDL particles [6]. In this study, EPA or LA significantly reduced the gene expression of SR-A1 and CD36, and EPA further increased the expression of ABCA1. These alterations corporately contributed to the less cholesterol uptake and more cholesterol efflux. Though lipid-poor apoAI is not synthesized by macrophages, we found PUFAs could up-regulate the mRNA level of apoAI. It is worth noting that the higher expression of apoAI increased cholesterol efflux in macrophages [18]. The protective effects of apoAI can also be attributed to its anti-inflammatory and antioxidative properties, not directly related to plasma HDL cholesterol levels [19]. NEFA-induced lipid accumulation in foam cells may be caused by increased lipid influx mediated by CD36. The up-regulation of ABCA1 was accompanied by the increased cholesterol efflux to apoAI and the reduced intracellular lipid content. These findings suggested that ABCA1 played a key role in regulating intracellular lipid accumulation. The ABCA1 appears to be the most effective receptor in mediating cholesterol efflux to apoAI. The half transporter ABCG1 also, to some extent, facilitates cholesterol efflux to HDL as acceptor [20-22]. The elevated cholesterol efflux to apoAI in medium rather than to HDL in EPA group supported the finding that EPA may induce more cholesterol efflux through ABCA1 pathway. But the lower fold-increase of ABCA1 in LA group indicated that there might be other pathways participating in the modulation of cholesterol efflux. But the changes of ABCA1 and ABCG1 in protein level have not been investigated in our experiment. Further study in this area is needed.

CIDE family consists of Cidea, Cideb and Cidec/Cide3. They play important roles in lipid storage, LD formation and lipolysis [23]. But their expression profiles and functions in AS is still unclear. Cidea protein, surrounding LDs and co-localizing with Perilipin, can efficiently repress lipolysis in human adipocytes [24,25]. Cidec inhibits lipolysis, promotes TG accumulation and contributes to the stability of formed LDs [8,13,24]. Fold-increase of Cidea and Cidec in PUFAs pre-treated foam cells may be helpful to promote TG accumulation and inhibit lipolysis [3,26]. Thus PUFA may exert its protective role by reducing the lipotoxicity in foam cells. Our previous study showed that the down-regulation of Cideb was closely related to the enhanced hepatic fatty acid oxidation [27]. It is certain that Cideb plays an important role in affecting lipid metabolism in foam cells, though whether Cideb could enhance lipid oxidation or lipolysis still needs further investigation.

PAT family is composed of five members: Perilipin, ADRP, TIP47, S3-12 and LSDP5. Perilipin and ADRP are constitutive proteins on LD membranes [28,29]. ADRP participates in LD formation in macrophages and foam cells [30] and facilitates cholesterol efflux from macrophages [31]. The increased ADRP and Perilipin in PUFA pre-treated group may help to reduce the toxicity of NEFA and contributes to the synthesis of lipid (including TG). Nascent LDs are predominantly decorated by TIP47 and S3-12 [17]. As lipid droplet coats, these two proteins are interchangeable and are necessary for TG storage [5,30]. LSDP5 is a PPARγ-induced LD-associated protein which can promote the fatty acid positioning. The expression of LSDP5 is also associated with the increased TG accumulation, for it can promote expansion of the storage pool of lipid substrates [28,32]. In conclusion, PUFAs have positive effect on maintaining TG content, reducing TC and CE contents in LDs. These effects may be closely related to the separate or combined expression of CIDE and PAT members. The meaningful alterations of these LD-associated proteins in mRNA level may provide us new ideas to explore lipid metabolism in foam cells.

Although PUFAs could not completely resist lipid deposition in foam cells, it can effectively reduce the accumulation of cholesterol. Several factors related to lipid metabolism in foam cells have been observed in this study, but the cause-effect studies have not been thoroughly conducted and further investigation is needed.

This study addressed that PUFAs pre-treated macrophage-derived foam cells showed dynamic gene changes. The expression of PPARγ and LD-associated proteins in transcriptional level can be, at least in part, linked to the process of LD formation. In summary, the different effects of NEFAs on TG and cholesterol flux in foam cells depend on an integrated function of relevant genes. We believe that the gene expression profiles of CIDE and PAT members should also be used as important indicators of LD metabolism in foam cells. More detailed researches should be performed in future to clarify the mechanism of lipid accumulation in foam cells.

## Materials and methods

### Materials

Human monocytic leukemia THP-1 cells were kindly donated by Dr. Bo-quan Jin (Department of Immunology, Fourth Military Medical University, Shaanxi, China). Human buffy coats were purchased from blood bank of Xijing Hospital (Fourth Military Medical University, Shaanxi, China). Whole human HDL (density = 1.063-1.021, containing HDL_2_ and HDL_3_) was purchased from Union Medical College (Beijing, China). Unless otherwise specified, all other reagents were purchased from Sigma-Aldrich (Shanghai, China).

### Monocyte isolation

A two-step procedure was used to isolate monocyte from buffy coats. Firstly, peripheral blood mononuclear cells (PBMCs) were isolated from buffy coats using a Ficoll-Hypaque gradient (density = 1.070). Then a slight hyperosmolar Percoll gradient (density = 1.064) was used for monocyte isolation [33]. Percoll solutions were prepared as follows: first an isosmotic Percoll was prepared by mixing one volume NaCl 1.5 M with nine volumes of Percoll (density = 1.130). Then the Percoll gradient was done by mixing isosmotic Percoll with PBS/Citrate (NaH_2_PO_4_ 1.49 mM; Na_2_HPO_4_ 9.15 mM; NaCl 139.97 mM; C_6_H_5_Na_3_O_7_.2H_2_O 13 mM; pH 7.2) in a 1: 1 (v/ v) ratio. Both gradients were centrifuged at 25-35°C, 400 g for 35 min. The purity of monocytes after the Percoll gradient isolation was higher than 85%. Platelets were eliminated by low speed centrifugation (100 g) after the Percoll gradient isolation.

### Cell culture

THP-1 cells and human peripheral monocytes were cultured in RPMI-1640 medium (Gibco, US) supplemented with 10% fetal bovine serum (FBS, Gibco, US), penicillin (100 U/ml) and streptomycin (100 μg/ml) in CO_2_/O_2_ incubator at 37°C. Then cells were incubated with 100 ng/ml phorbol 12-myristate 13-acetate (PMA) for 24 hours to facilitate macrophage formation. The adherent macrophages were washed three times with phosphate-buffered saline (PBS) and incubated with cell culture medium containing 2% FBS for another 24 hours with or without NEFA. After the incubation, macrophages were treated with 50 μg/ml ox-LDL for another 48 hours for foam cell formation.

### Toxicology assay

The THP-1-derived macrophages were treated by different NEFAs separately with time and dose gradients. The cell viability was tested using 3-(4,5-Dimethylthiazol-2-yl)-2,5-diphenyltetrazolium bromide (MTT) colorimetric assay as previously described [34]. THP-1 macrophages were separately cultured with NEFAs in a final concentration from 50 μM to 200 μM in 96-well micro plates for 24 hours to 96 hours. After an appropriate incubation (37°C, 4 hours with 20 μl MTT (5 mg/ml) in each well), 150 μl dimethyl sulfoxide (DMSO) was used to replace the medium containing MTT, and then plates were shook for 10 minutes. Absorbance at 490 nm was measured. Each experiment was independently repeated three times. Bovine serum albumin (BSA) mixed with DMSO and PBS was set as the control.

### Isolation and oxidation of LDL

Native LDL (density = 1.020-1.063) were isolated from human plasma by sequential flotation ultracentrifugation at 60,000 rpm (24 hours, 10°C). Before oxidative modification, the LDL was dialyzed against PBS, filtered through a 0.2 μm millipore membrane and stored in PBS containing 10 mM EDTA at 4°C. LDL oxidation was induced by 40 mM HOCl (corresponding to oxidant/protein molar ratios of 2000/1) for 30 min at 37°C. Ox-LDL was dialyzed overnight against saline (0.15 M NaCl and 300 μM EDTA, pH 7.4). The protein concentration was determined by methods of Lowry [35].

### NEFA pre-treatment

Macrophages were exposed to NEFAs at a final concentration of 100 μM which was within the physiologic plasma range [32,36]. After 24 hours incubation, 50 μg/ml ox-LDL was added. Macrophages were incubated for another 48 hours to induce foam cells formation. Each NEFA was conjugated by fatty acid free BSA in a 4:1 molar ratio at 37°C for at least 1 hour prior to treatment as previously described [37]. NEFA was added to the culture medium with a minimal volume (< 0.1%) of DMSO. Three independent experiments with triplicate samples (each sample with 2.5 × 10^6^ cells/60 mm dish) were performed for each test.

### Cholesterol loading and efflux

After treatment with PMA and NEFA, THP-1 macrophage were washed with PBS and incubated with 50 μg/ml ox-LDL, 1 μCi/ml [^3^H] cholesterol and 2 mg/ml BSA for 40 hours. After 8 h equilibration with MEM containing 1 mg/ml of BSA, macrophages were washed twice with PBS and treated for 12 h with serum-free medium containing: (1).2 mg/ml BSA; (2).2 mg/ml BSA and 15 μg/ml lipid-free human apoAI; (3).2 mg/ml BSA and 50 μg/ml of human HDL. Then the supernatant was collected and centrifuged at 12 000 rpm for 15 min to remove debris. Cells were lysed with 0.5 ml of 0.1 N NaOH. The radioactivity in both the supernatant and cellular lipid was measured by scintillation counting. Background efflux was determined by control cells incubated with 2 mg/ml BSA. Human HDL was prepared as previously described [38,39]. The data were normalized by total [^3^H]-cholesterol radioactivity in supernatant and cell pellet.

### Real time quantitative PCR assay

The targeted genes, primer sequences and Genebank number for real-time PCR assays performed in this study are summarized in Table 2. Total RNA was extracted from macrophages and foam cells (THP-1 cell line) with Trizol reagent (Invitrogen, US) and then reverse transcribed to cDNA in a final volume of 30 μl. Real-time PCR assays were performed with an iCycler IQ Real Time PCR Detection System (BIO-RAD,US) and the SYBR Prime Script RT-PCR Kit (Takara Dalian, China). 5 pmol primer and 100 ng cDNA were used in a final volume of 25 μl. Homo sapiens hypoxanthine phosphoribosyl transferase 1 (HPRT1) was used as a housekeeping gene for its stable expression in monocytes and macrophages. The PCR amplification program was 95°C for 3 minutes, followed by 45 cycles of 95°C for 30 seconds (s), 60°C for 30 s and 72°C for 30 s. All samples were tested three times.

**Table 2:** Real-time PCR primer sequences

**Gene**	**Primer sequences**	**Genebank number**
*SR-A1*	Forward: CCAGGGACATGGGAATGCAA	NM_138715.2
	Reverse: CCAGTGGGACCTCGATCTCC	
*CD36*	Forward: GAGAACTGTTATGGGGCTAT	NM_001001548.2
	Reverse: TTCAACTGGAGAGGCAAAGG	
*ABCA1*	Forward: GCACTGAGGAAGATGCTGAAA	NM_005502.2
	Reverse: AGTTCCTGGAAGGTCTT GTTC	
*ApoAI*	Forward: TGTGTCCCAGTTTGAAGGC	NM_000039.1
	Reverse: CTCCTTTTCCAGGTTATCCCAG	
*PPARγ*	Forward: CACAAGAACAGATCCAGTGGTTGCAG	NM_005037.5
	Reverse: AATAATAAGGTGGAGATGCAGGCTCC	
*Cidea*	Forward: GGGATCACAGACTAAGCGAG	NM_001279.3
	Reverse: TGACGAGGGCATCCAGAG	
*Cideb*	Forward: TGATGGTGTTGCAGTCTGG	NM_014430.2
	Reverse: AAAGAGGTCTCGAGGGTTTTG	
*Cidec*	Forward: TTGATGTGGCCCGTGTAACGTTTG	NM_022094.2
	Reverse: AAGCTTCCTTCATGATGCGCTTGG	
*Perilipin*	Forward: GCCATGTCCCTATCAGATGC	NM_001145311.1
	Reverse: GTTGTCGATGTCCCGGAATT	
*ADRP*	Forward: CTGAGCACATCGAGTCACATACTCT	NM_001122.2
	Reverse: GGAGCGTCTGGCATGTAGTGT	
*TIP47*	Forward: GCTGGACAAGTTGGAGGAGA	NM_001164189.1
	Reverse: CCGACACCTTAGACGACACA	
*S3-12*	Forward: ACATCTTCCACCCCATGAATG	NM_001080400.1
	Reverse: GTGTTCAAATGCCCGCTG	
*LSDP5*	Forward: AGCACGATGTCTGAAGAAGAG	DQ_839131.1
	Reverse: TCCTTGGCTGCACTGTAAAC	
*HPRT1*	Forward: TGACACTGGCAAAACAATGCA	NM_005502.2
	Reverse: GGTCCTTTTCACCAGCAAGCT	

### Lipid analysis

Cells were fixed in 4% paraformaldehyde for 30 minutes, and then washed three times with PBS before incubated in a working solution of Nile Red. Nucleus of the cell was dyed with Hoechst 33258 as previously described [8]. The image of lipid staining was captured and quantified by Olympus Type BX51TRF microscope (Olympus Co., Japan) and Image-Pro Plus 6.0 software (Media Cybernetics, US). Cell lipids were extracted with hexane: isopropanol at 3:2 (vol: vol). The quantities of cellular TG, TC and free cholesterol were determined by enzymatic colorimetric assays using commercial reagents (Wako Pure Chemical Industries, Japan) according to the manufacturer’s protocol. The concentration of CE was determined by subtracting free cholesterol from TC. After lipid extraction, residual proteins were used for protein quantitative determination.

### Data presentation

A transformed ratio was used to calculate fold-change of mRNA in the process of macrophages differentiation into foam cells. The fold-change of mRNA in foam cells vs. in macrophages was calculated as: log_2_(mRNA level in foam cell/mRNA level in macrophage). We specified the value of log_2_(mRNA level in foam cell control/mRNA level in macrophage control) as the control for all the samples in a particular study. The baseline was set to be Ratio (logarithm scale) = 0, which means that bars with ratios greater than one (increase) pointed up, and ratios smaller than one (decrease) pointed down.

### Statistics

Results were expressed as mean ± SEM. Differences between groups were determined using one way analysis of variance (ANOVA). Dunnett’s-*t* test was used for a multiple comparison. *P* < 0.05 was taken as significance.

## Abbreviations

PUFA: Polyunsaturated fatty acid; LD: Lipid droplet; NEFA: Non-esterified fatty acid; SFA: Saturated fatty acid; PA: Palmitic acid; MUFA: Monounsaturated fatty acid; OA: Oleic acid; LA: Linoleic acid; EPA: Eicosapentaenoic acid; CIDE: Cell death-inducing DFF45 like effector; PAT: Perilipin-Adipophilin-TIP47; AS: Atherosclerosis; PBMC: Peripheral blood mononuclear cell.

## Competing interests

There are no conflicts of interest in this study.

## Authors’ contributions

YS, LJZ and HL carried out the study design, data collection and analysis, and drafted the manuscript. FFL participated in the cell-based experiments and review of the manuscript. YG and FL helped conduct the real-time PCR experiment. LNJ helped perform the isolation of ox-LDL. JY and QL were responsible for the study design, the funding, the data analysis, and the manuscript draft. All authors read and approved the final manuscript.

## Supplementary Material

Additional file 1: Figure S1 Specific mRNA expressions of proteins with transcription regulated by PPARγ in NEFA pre-treated macrophages and foam cells. Data represent mean ± SEM (n = 3). ^※^*P* < 0.05 vs. the value in macrophages; ^†^*P* < 0.05 vs. the macrophage control; ^‡^*P* < 0.05 vs. the foam cell control. Click here for file

Additional file 2: Figure S2 Specific mRNA expressions of LD-associated proteins in NEFA pre-treated macrophages and foam cells (**A**.CIDE family members; **B**. PAT family members). Data represent mean ± SEM (n = 3). ^※^*P* < 0.05 vs. the value in macrophages; ^†^*P* < 0.05 vs. the macrophage control; ^‡^*P* < 0.05 vs. the foam cell control. Click here for file
