# Supplementary figures and images for: Polyunsaturated fatty acid relatively decreases cholesterol content in THP-1 macrophage-derived foam cell: partly correlates with expression profile of CIDE and PAT members
Source: Lipids Health Dis. 2013 Jul 23;12:111. doi: 10.1186/1476-511X-12-111 (PMC3751560; doi:10.1186/1476-511X-12-111)

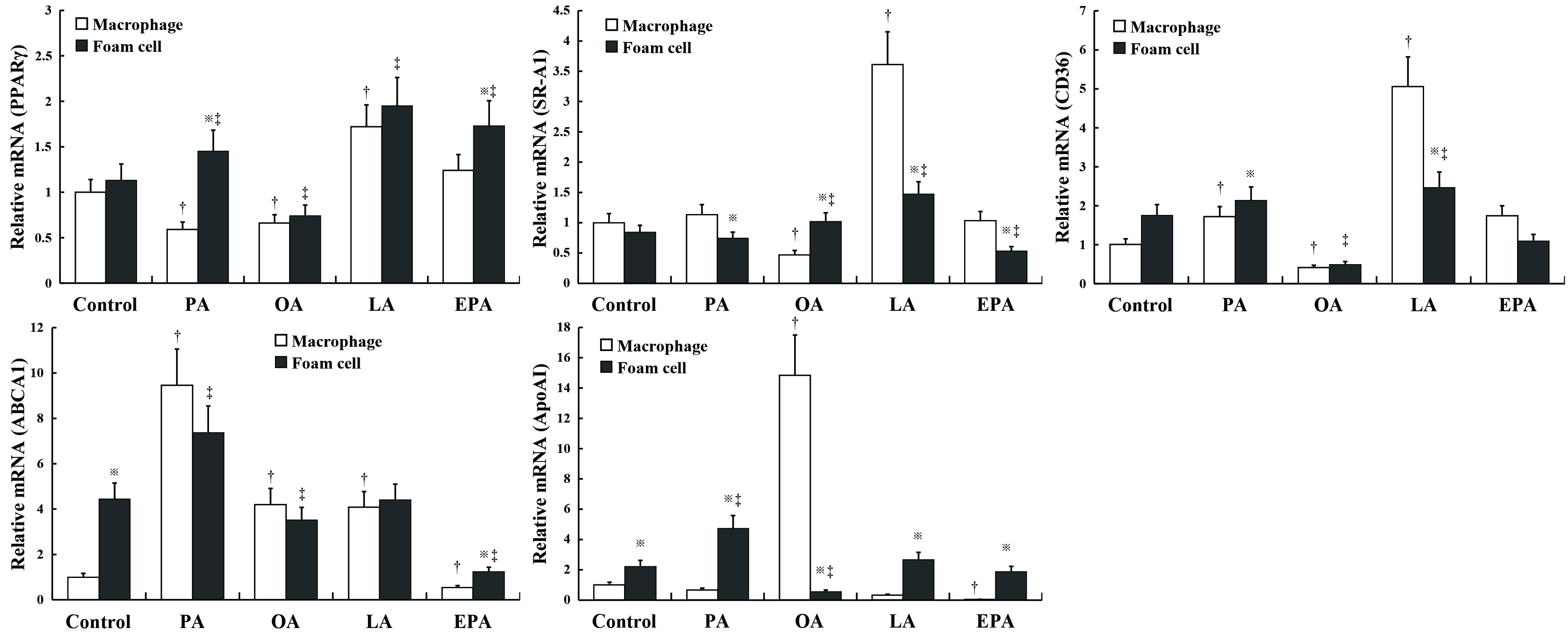

Supplement: Additional file 1: Figure S1 — Specific mRNA expressions of proteins with transcription regulated by PPARγ in NEFA pre-treated macrophages and foam cells. Data represent mean ± SEM (n = 3). ※P < 0.05 vs. the value in macrophages; †P < 0.05 vs. the macrophage control; ‡P < 0.05 vs. the foam cell control. [file 1476-511X-12-111-S1.jpeg]

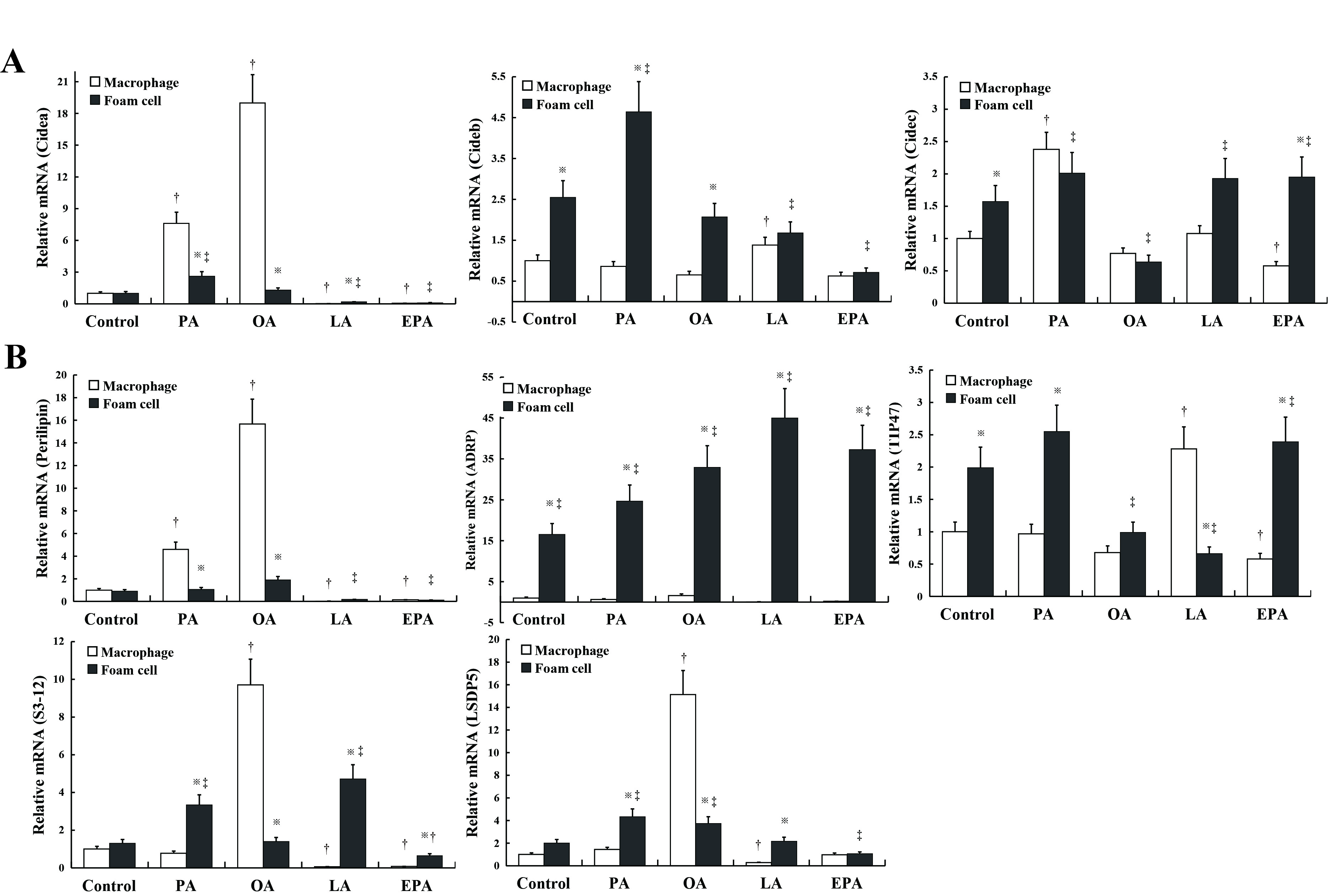

Supplement: Additional file 2: Figure S2 — Specific mRNA expressions of LD-associated proteins in NEFA pre-treated macrophages and foam cells (A.CIDE family members; B. PAT family members). Data represent mean ± SEM (n = 3). ※P < 0.05 vs. the value in macrophages; †P < 0.05 vs. the macrophage control; ‡P < 0.05 vs. the foam cell control. [file 1476-511X-12-111-S2.jpeg]
